# Genomic epidemiology and antimicrobial resistance profile of Shigella isolated from diarrhoea diseases in under-five children in Blantyre, Malawi

**DOI:** 10.1099/mgen.0.001838

**Published:** 2026-09-11

**Authors:** Grace Thandekire Sibande, Charlotte E. Chong, Anmol Kiran, Belson Kutambe, Mary Charles, Victor Maiden, Kate S. Baker, Jennifer Cornick

**Affiliations:** 1 Malawi-Liverpool-Wellcome Programme, Blantyre, Malawi; 2 Department of Clinical Infection, Microbiology and Immunology, Institute of Infection, Veterinary and Ecological Sciences, University of Liverpool, Liverpool, UK; 3 Department of Genetics, Applied Microbial Genomics Laboratory, University of Cambridge, Cambridge, UK; 4 School of Biochemistry and Cell Biology, University College Cork, Western Rd, Cork, T12 CY82, Ireland

**Keywords:** antimicrobial resistance, genomic epidemiology, *Shigella*, whole-genome sequencing

## Abstract

Antimicrobial resistance (AMR) in *Shigella* is rising globally, complicating shigellosis management. Whole-genome sequence analysis (WGSA) has advanced our understanding of AMR and transmission dynamics, yet contemporary whole-genome sequencing data from *Shigella* in sub-Saharan Africa remain scarce. In this study, we applied WGSA to 27 *Shigella* isolates collected from children presenting with diarrhoea at Ndirande Health Centre in Malawi (2022–2023), as part of the Enterics for Global Health *Shigella* surveillance study. Serotyping, AMR profiling and phylogenetic analysis revealed *Shigella sonnei* as the dominant serogroup, with distinct genetic clustering relative to global reference strains among *S. sonnei*, *Shigella flexneri* and *Shigella boydii*. We identified 16 AMR genes linked to ten antimicrobial classes with *qnrS1* and *qnrB19* genes conferring resistance to fluoroquinolone, alongside IncFIB(K) and IncFII plasmid replicon markers. Importantly, no azithromycin resistance determinants were detected both genotypically and phenotypically, providing baseline evidence that warrants continued surveillance of current first-line treatment. However, the detection of fluoroquinolone resistance genes with plasmid replicon markers in the absence of phenotypic resistance might indicate a silent reservoir with epidemic potential. This is the first contemporary *Shigella* data from a large-scale diarrhoea disease surveillance study in Malawi, providing essential baseline information for guiding antibiotic treatment and future vaccine development efforts, contributing to the efforts to combat shigellosis in Malawi and other similar regions.

**Impact Statement**

Fluoroquinolone-resistant *Shigella* is an escalating global health threat, particularly in low- and middle-income countries (LMICs) where shigellosis remains a leading cause of childhood diarrhoeal mortality. In Malawi – a high-burden but understudied region – data on circulating *Shigella* species, resistance patterns and outbreak dynamics are limited. Available information came from only eight isolates recovered from children with medically attended diarrhoea, with a single isolate reported to harbour genotypic determinants of fluoroquinolone resistance (FQR). Given the sparse baseline data, the potential emergence of FQR *Shigella* strains could undermine current treatment strategies.

In this study, all Malawian *Shigella* isolates (*n*=27) analysed were multidrug-resistant, with 22% (*n*=6) harbouring FQR genes (*qnrS1/qnrB19*) detected alongside plasmid replicon markers, previously linked to global outbreaks. Importantly, no azithromycin resistance was detected, providing baseline evidence that warrants continued surveillance of current first-line treatment. However, in the absence of phenotypic FQR, the detection of putative plasmid-associated resistance elements likely reflects their limited standalone effect and regulatory constraints, consistent with a silent genetic reservoir that may facilitate rapid resistance emergence and dissemination across strains and species under antibiotic selective pressure.

The emergence and global dissemination of epidemic clones within species, such as *Shigella sonnei* and *Shigella flexneri*, enriched with virulence factors and conserved antimicrobial resistance (AMR) cassettes, underscores their latent dual threat as drivers of both disease severity and resistance spread. These findings highlight an urgent need for proactive surveillance to detect silent resistance reservoirs and tailored interventions, such as vaccines targeting prevalent serotypes to reduce antibiotic reliance.

This study underscores the critical importance of genomic surveillance in under-resourced regions, where undetected AMR mechanisms can fuel regional and global AMR crises. Addressing this threat requires integrated strategies: antimicrobial stewardship to curb misuse, molecular tracking of mobile resistance elements and targeted vaccine deployment. By prioritizing these efforts, Malawi and similar LMICs can mitigate the devastating convergence of shigellosis and AMR, safeguarding both local populations and global health security.

## Data Summary

The authors confirm all supporting data, details of bioinformatic packages and protocols have been provided within the article or through supplementary data files. The raw sequencing reads from the 27 *Shigella* isolates which were subjected to whole genome sequencing have been deposited in the National Center for Biotechnology Information (NCBI) Short Read Archive under Bioproject accession number: PRJNA1219651. Detailed metadata for each isolate, including SRA accession numbers are provided in Table S1. Reference *Shigella* isolate accession numbers are also cross-referenced with their metadata in Table S2.

## Introduction

*Shigella* species are the leading bacterial cause of moderate-to-severe (MSD) diarrhoea in under-five children in resource-poor settings, with the highest burden in sub-Saharan Africa and Southern Asia, where *Shigella* account for one-third of diarrhoeal deaths [1–3]. The multi-country Antibiotics for Children with Severe Diarrhoea study identified *Shigella* as a leading cause of MSD, detecting it in 12.6% of 6,692 cases [4]. While supportive care remains a cornerstone of treatment for *Shigella* diarrhoea, the World Health Organisation (WHO) recommends antimicrobial therapy for all clinically suspected cases of *Shigella dysenteriae* type 1 to reduce mortality and long-term complications [5]. WHO guidelines recommend ciprofloxacin as a first-line treatment for shigellosis and pivmecillinam, ceftriaxone or azithromycin (AZM) as a second-line option [6]. Meanwhile, Malawi’s national treatment guidelines prioritize AZM as the first-line treatment, with ciprofloxacin as the second-line [7]. Antimicrobial resistance (AMR) is a growing concern across all *Shigella* species, but *Shigella sonnei* has emerged as a major driver of fluoroquinolone resistance (FQR) [6, 8].

The genus *Shigella* comprises four species: *Shigella boydii, S. dysenteriae, Shigella flexneri* and *S. sonnei*, alongside ∼50 serotypes, highlighting the considerable diversity of the pathogen [9]. This diversity and serotype-specific immunity linked to the lipopolysaccharide O antigen complicates vaccine development contributing to the absence of a licensed human *Shigella* vaccine [9–11]. Consequently, current strategies are focused on multi-valent serotype-based approaches to provide broad protection against diverse *Shigella* serotypes [10]. Given the AMR crisis, the WHO prioritizes the development of vaccines targeting fluoroquinolone-resistant *S. sonnei,* a major AMR organism [11]. In low- and middle-income countries (LMICs), the gut microbiome serves as a reservoir for plasmid-mediated AMR, driven by unregulated antimicrobial use [12]. Whole-genome sequencing analysis (WGSA) is instrumental in supporting control efforts by identifying the genetic basis of serotype diversity, tracking the emergence of AMR and informing vaccine design [13].

Our understanding of the AMR profile and species/serotype distribution of *Shigella* in Malawi is limited. Previously, WGSA was used to characterize eight *Shigella* isolates recovered from children with medically attended diarrhoea in Malawi and reported only a single isolate that had genotypic determinants of FQR [8].

However, these isolates were recovered over a decade ago, and it is unclear if this small dataset, which did not include any *S. sonnei*, the second-most common cause of shigellosis in LMIC settings, is representative of the current *Shigella* population in Malawi [8]. In this study, we apply WGSA to 27 *Shigella* isolates recovered from children with medically attended diarrhoea, recruited to the Enterics for Global Health (EFGH) *Shigella* Surveillance Study carried out during 2022–2023. We characterize the *Shigella* genotype distribution and AMR determinants. These updated data provide baseline genomic and AMR information that can inform local treatment guidelines and public health interventions, including vaccine development efforts to reduce the burden of shigellosis in Malawi and similar settings.

## Methods

### Dataset, bacterial isolates and sequencing

EFGH was a hybrid surveillance study of children aged 6–35 months presenting with diarrhoea to Ndirande Health Centre, Blantyre, Malawi [13]. Between 3 August 2022 and 31 July 2023, 1,399 children were recruited to the EFGH study, of which 3% (44/1,399) had confirmed shigellosis via microbiological culture and biochemical testing, followed by serotyping using gold standard latex agglutination. Molecular typing and speciation were performed using TaqMan array card, as described in detail previously [14]. DNA was extracted from the 44 microbiologically confirmed *Shigella* isolates using the MagMax^TM^ CORE Nucleic Acid Purification Kit (Thermo Fisher), operated on the KingFisher^TM^ Flex system, per protocol. This method efficiently isolated DNA suitable for downstream molecular speciation and sequencing, with only two isolates failing quality control. Isolates underwent WGS on the Malawi-Liverpool-Wellcome Programme Illumina MiSeq in a single batch, following in-house protocols (42/44). Sequencing generated paired-end reads of 151–251 bp, reflecting minor trimming variability. For phylogenetic context, an additional 124 publicly available genomes, selected from a previously published phylogenetic study of Malawian *Shigella* isolates were included [8]. These comprised 118 *Shigella* genomes representing diverse global lineages and six *Escherichia coli *isolates for comparison. Accession numbers and metadata for all genomes, including both EFGH study isolates and publicly available genomes, are provided in Table S2.

### Genome assembly, annotation and *in silico* serotyping

All raw sequence reads were quality-trimmed using Trimmomatic (v0.39) [15]. Adapter sequences were removed using ILLUMINACLIP:TruSeq3-PE.fa:2 : 30 : 10, low-quality bases were trimmed using a sliding window of four bases and an average quality threshold of 20 (SLIDINGWINDOW:4 : 20), leading and trailing bases with quality below 3 were removed (LEADING:3 TRAILING:3) and reads shorter than 50 bp were discarded (MINLEN:50). Sequence quality was assessed both before and after trimming using FastQC (v0.12.1) and MultiQC (v1.0) [16, 17].

To be included in subsequent analyses, isolate reads were required to meet predefined quality criteria used in our bacterial WGS quality-control pipeline isolate reads: per-base mean phred score ≥28, even per-base sequence content, smooth per sequence G+C content curve with peak G+C content between 50 and 51 mol%, per base N content <5, <5% overrepresented sequences and <5% sequences with adapter contamination. Reads with residual adapter sequence were retained, as this low level of contamination is unlikely to affect downstream analyses.

Draft genomes were assembled from quality-trimmed sequence reads using Unicycler (v0.5.0) in default mode, with a minimum contig length of 150 bp and using eight threads [18]. Genome annotation was performed using Bakta (v1.9.2) with the Bakta full reference database (v5.1) [19]. Genome assembly quality was assessed using Quast (v5.2.0) [20]. *In silico* confirmation of *Shigella* or Enteroinvasive *E. coli* and serotype prediction on the assemblies was carried out using ShigEifinder (v1.3.5) with 100% sensitivity on assembled genomes [21]. Taxonomic classification of the sequenced isolates was further confirmed using Kraken-2 (v2.1.2) and any isolates were retained if ≥90% of reads were classified as *Shigella* by Kraken-2 [22].

### Sequence mapping, variant calling and phylogenetic reconstruction

Core-SNP alignments were generated from quality-trimmed sequence reads using Snippy (v4.6.0) mapping the sequence reads to the complete *S. flexneri 2a strain 301* reference genome (NCBI Reference Sequence: NC_004337.2) [23]. This reference was selected because it is a complete, well-annotated *Shigella* genome that provides a consistent framework for comparative analysis across the study isolates. Recombinant regions were identified and removed using Gubbins (v3.3.1) [24]. Read mapping quality was assessed using Qualimap (v2.3) [25].

A maximum likelihood phylogeny was constructed using RAxML-HPC (v8.2.12) under the GTR+G nucleotide substitution model, with 100 rapid bootstrap replicates on the recombinant-filtered core SNP alignment [26]. *E. coli K-12 MG1655 M2 (ED1a-119*) (NCBI Reference Sequence: GCF_000026305.1) was included as an outgroup to root the phylogenetic tree. The resulting phylogenetic tree was visualized using the Interactive Tree of Life [27].

### Phenotypic profiling, detection of AMR genetic determinants and mobile genetic elements

Phenotypic antimicrobial susceptibility testing was performed as part of the broader EFGH surveillance study using the Kirby–Bauer disc diffusion method, with susceptibility interpreted according to Clinical and Laboratory Standards Institute (CLSI) breakpoints, as described in detail previously [14]. *In silico* identification of AMR and virulence profiles was performed using AMRFinderplus (v3.12.8) and VirulenceFinder (v2.0.4) [28]. Plasmid content was assessed by comparing all assembled contigs against the PlasmidFinder webtool (v2.1.6) which compares assembled contigs against the Plasmid Finder database using blastn [29]. Only hits with ≥95% sequence identity and ≥60% coverage were considered putative plasmid contigs. A particular focus was placed on detecting Inc(F) incompatibility group plasmids, due to their known association with multidrug-resistant plasmids (Table S5). For comparative analysis of AMR regions and visualization of plasmid and contig alignments, the Artemis Comparison Tool (v18.0.0) was used [30].

## Results and discussion

### Serotyping and global genomic diversity

Of the 42 isolates sequenced, 27 were confirmed by Kraken-2 as *Shigella*, 1 as *E. coli* and 14 as *Providencia alcalifaciens*. Among the 27 isolates included in our analysis, aligned read bases coverage ranged from 1.6 to 4.3 Mbp, corresponding to 33–90% of the 4.8 Mbp reference genome, with a median coverage of 3.67 Mbp (77%). The variation in genome coverage likely reflects the genetic diversity of the study isolates, which included multiple *Shigella* species mapped against a single reference genome. Consequently, more genetically divergent isolates exhibited lower mapping coverage, which may have reduced SNP resolution. In terms of species distribution, 37% (*n*=10/27) were *S. sonnei,* 33% (*n*=9/27) were *S. flexneri* and 30% (*n*=8/27) were *S. boydii* (Table S3). We did not identify any *S. dysenteriae* in our dataset, which is consistent with regional epidemiological trends [31]. *S. dysenteriae*, particularly serotype 1, is more commonly associated with severe outbreaks in specific settings, such as South Asia and East Africa, rather than endemic transmission in Southern Africa, including Malawi [31]. The dominance of *S. sonnei* and *S. flexneri* in our dataset*,* with proportions not markedly higher than *S. boydii,* may reflect genuine epidemiological trends or could be influenced by the relatively small sample size. This dataset represents all WGS-confirmed *Shigella* cases detected from the EFGH study. The finding also aligns with previously reported prevalence trends in sub-Saharan Africa, where *S. flexneri* has historically been prevalent until recently where *S. sonnei* has now become dominant, likely due to improvement in water quality and sanitation [31, 32]. Serotype distribution varies by region and study population, but notably, the Vaccine Impact on Diarrhoea in Africa study – a large, multi-country investigation reported a predominance of *S. flexneri* (67.6%; *n*=243/359) with considerable variations in circulating serotypes [32]. Broader and more representative patterns may only become apparent once the full EFGH surveillance dataset is analysed.

Comparison of speciation and serotyping results from WGSA using ShigEifinder against the gold standard conventional microbiological identification and serotyping methods showed 96% (*n*=26/27) concordance, underscoring the reliability of ShigEifinder as a genomic tool for accurate *Shigella* speciation (Table S3). We identified two *S. flexneri* serotypes in our dataset: *S. flexneri* 6 (4/9) and *S. flexneri* Xv (*4 c*) (5/9) using WGSA (Table S2). Interestingly, all the isolates typed as *S. flexneri Xv (4 c*) using WGSA were typed as *S. flexneri 4A* via latex agglutination and as *S. flexneri X* using TAC (Table S3). This discordance highlights limitations in typing methods; for instance, *S. flexneri 4A* was identified through latex agglutination, but WGS-based serotype prediction indicated *S. flexneri* Xv (*4 c*), a subtype not typically detected by conventional serotyping panels. If latex serotyping alone had been used, this could have led to an overestimation of *S. flexneri 4* a burden in our population. *S. flexneri* Xv (*4 c*) has been reported as multidrug-resistant (MDR) in China, raising important public health concerns [32–34]. However, we did not observe MDR among these strains in our dataset. These findings underscore the importance of complementary typing, as both methods have limitations. Improved understanding of serotype distribution and typing discrepancies through larger surveillance studies will be essential to support *Shigella* vaccine development and accurately prioritize prevalent strains. Based on WGSA typing, a candidate quadrivalent vaccine formulation under development, comprising *S. flexneri 2a, 3a, 6 and S. sonnei,* would cover ∼48% (*n*=13/27) of the isolates in our dataset, while an alternative quadrivalent formulation (*S. flexneri 1b, 2a, 3a and S. sonnei*) would cover 33% (*n*=9/27), highlighting gaps in vaccine coverage against the circulating *Shigella* population [35].

**Fig. 1.**
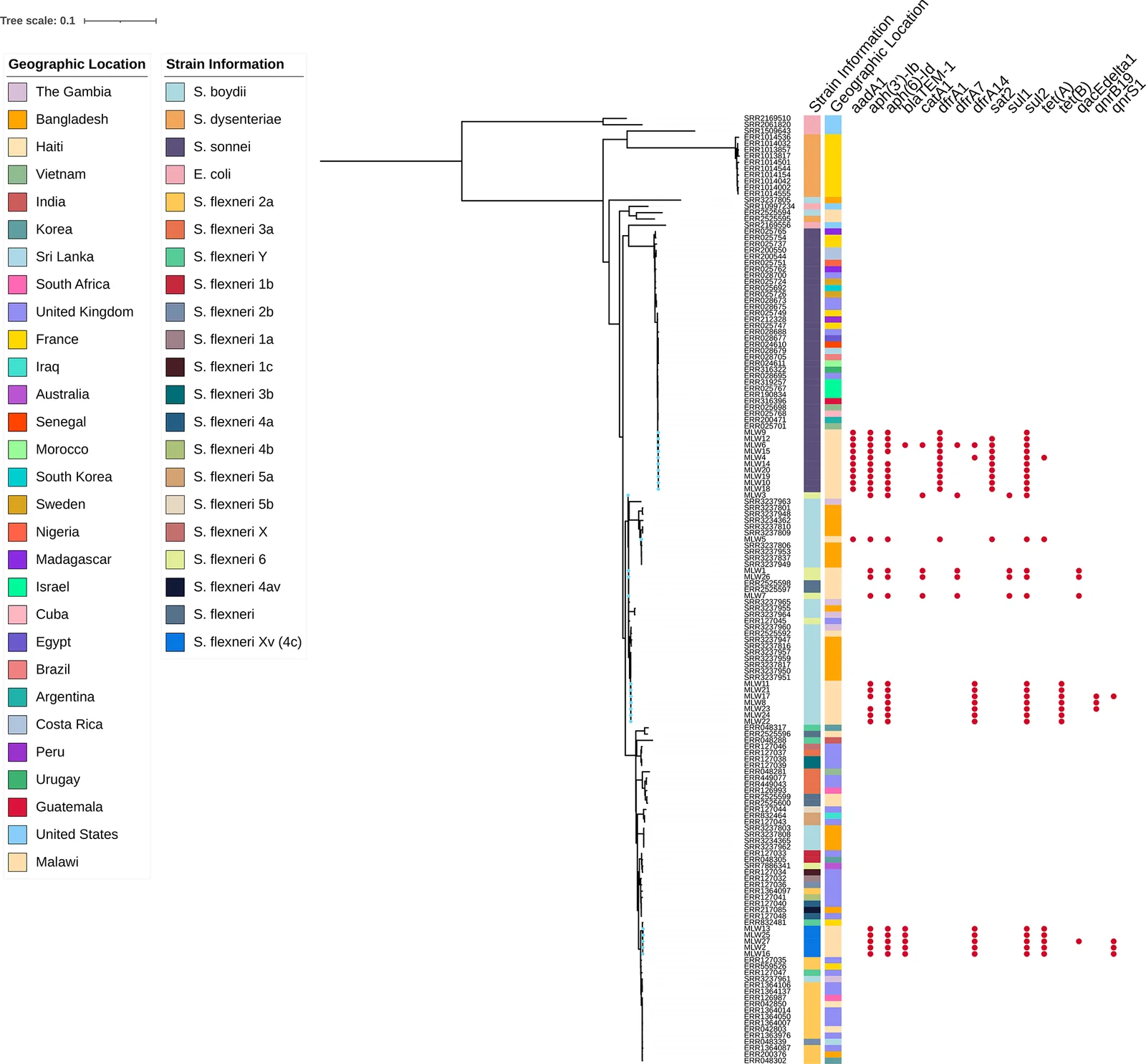
Maximum-likelihood phylogenetic tree of *Shigella* isolates from medically attended diarrhoea in Malawian children and 124 global isolates, as indicated by the inlaid key. Metadata columns to the right, from left to right, strain information, geographic location, AMR genes. AMR gene annotation was generated only for the Malawian isolates included in this study. The global comparator isolates were included solely to provide phylogenetic context and were not assessed for AMR determinants as part of the present analysis.

To explore the evolutionary history and global context of our isolates, we constructed a maximum likelihood phylogenetic tree inferred from 40,075 core SNPs, which revealed expected evolutionary trajectories among *Shigella spp*. (Fig. 1). Specifically, *S. flexneri and S. sonnei* were highly clonal, consistent with global patterns of rapid human-to-human transmission and epidemic spread [36, 37]. They were also more closely related to each other than with *S. dysenteriae* and *E. coli*, reflecting deeper evolutionary divergence between these species. Malawi isolates primarily formed monophyletic clusters within *S. sonnei* (*n*=10), *S. flexneri* (*n*=5) and *S. boydii* (*n*=7) with only five isolates, primarily *S. flexneri 6* falling outside of these three main clusters (Fig. 1). This likely reflects circulation of genetically diverse local lineages, as *S. flexneri 6* is less prevalent than dominant epidemic clones and its large phylogenetic distance from *S. flexneri Xv (4 c*) reflects deep evolutionary divergence, including differences in O-antigen structure and accessory genome content [38]. Notably, the *S. boydii* cluster showed varying levels of relatedness; five isolates were closely related (48–89 pairwise SNPs), whereas one isolate differed by ∼1,030–1,045 SNPs and another by 4,610–8,624 SNPs, suggesting local genetic diversification (Fig. 1, Table S4). This overall clustering supports sustained local circulation in this high-transmission setting [39]. The clusters specifically correlated with globally disseminated sequence types (STs), including ST152 (*S. sonnei*) and ST245 (*S. flexneri*), underscoring both local transmission networks and overlapping global ancestry (Fig. 1).

### AMR genotype, phenotype and virulence profiles

All isolates harboured genes conferring resistance to three or more drug classes, with conserved AMR profiles spanning nine antimicrobial classes. Promisingly, none of the isolates carried resistance determinants associated with all WHO-recommended antibiotics (ciprofloxacin, pivmecillinam, ceftriaxone or AZM) (Table 1) [6]. Resistance associated genes were frequently detected for sulfonamides (*sul1/sul2*), tetracyclines (*tet(A)/tet(B*)) and β-lactams (*blaTEM-1*) (Table 1). The recurrence of resistance-associated gene profiles, including *sul1/sul2*, *tet(A/B*) and *qacEdeIta1* among *S. sonnei* and *S. flexneri* isolates highlights the presence of shared AMR profiles among circulating lineages, some of which may be associated with plasmids (Table 1). These findings align with studies from LMIC settings showing that enteric bacterial populations can serve as reservoirs of plasmid-associated resistance determinants, where antimicrobial selection pressure may contribute to the persistence of stable MDR genotypes [12].

**Table 1.** Genotypic AMR determinants and phenotypic AMR profiles of *Shigella* isolates from Malawian children with medically attended diarrhoea

EFGH isolate	***Species***	***Contig length BP***	***Resistance gene***	***Genotypic resistance***	***Phenotypic resistance^1^***
MLW1	*S. flexneri 6*	21,993 *6,199*	sul1, qacEdelta1, dfrA7, catA1 *sul2, aph(3″)-Ib, aph(6)-Id*	Sulfonamide, quaternary ammonium, trimethoprim, phenicol sulfonamide, aminoglycoside	sulfamethoxazole–trimethoprim
MLW2	*S. flexneri Xv (4* c)	184,596* 2,486 *25,428*	*tet(A) dfrA14* *qnrS1, blaTEM-1, aph(6)-Id, aph(3″)-Ib, sul2*	Tetracycline trimethoprim quinolone, β-lactam, aminoglycoside, sulfonamide	Sulfamethoxazole–trimethoprim, ampicillin
MLW3	*S. flexneri 6*	6,141 *4,825* *4,820*	*aph(6)-Id, aph(3″)-Ib, sul2* *catA1* *sul1, qacEdelta1, dfrA7*	Aminoglycoside, sulfonamide phenicol sulfonamide, quaternary ammonium, trimethoprim	Sulfamethoxazole–trimethoprim
MLW4	*S. sonnei*	9,170 *5,683* *1,501* *1,217* *568*	*aadA1, sat2, dfrA1* *tet(A) aph(3″)-Ib, sul2* *aph(6)-Id dfrA14*	Aminoglycoside, streptothricin, trimethoprim tetracycline aminoglycoside, sulfonamide aminoglycoside trimethoprim	Sulfamethoxazole–trimethoprim
MLW5	*S. boydii*	33,643 *8,379*	*dfrA1, sat2, aadA1* *tet(A), aph(6)-Id, aph(3″)-Ib, sul2*	Trimethoprim, streptothricin, aminoglycoside tetracycline, aminoglycoside, sulfonamide	Sulfamethoxazole–trimethoprim
MLW6	*S. sonnei*	9,169 *8,412*	*dfrA1, sat2, aadA1* *sul2, aph(3″)-Ib, aph(6)-Id, tet(A*)	Trimethoprim, streptothricin, aminoglycoside sulfonamide, aminoglycoside, tetracycline	Sulfamethoxazole–trimethoprim
MLW7	*S. flexneri 6*	44,323 *8,710* *3,241*	*dfrA51* *sul1, qacEdelta1, dfrA7, catA1* *sul2, aph(3″)-Ib, aph(6)-Id*	Trimethoprim sulfonamide, quaternary ammonium, trimethoprim, phenicol sulfonamide, aminoglycoside	Sulfamethoxazole–trimethoprim
MLW8	*S. boydii*	6,768 *4,309* *2,700*	*sul2, aph(6)-Id, dfrA14* *tet(B) qnrB19*	Sulfonamide, aminoglycoside, trimethoprim tetracycline quinolone	Sulfamethoxazole–trimethoprim
MLW9	*S. sonnei*	8,406 *1,769* *1,126*	*sul2, tet(A), aph(6)-Id, aph(3″)-Ib aadA1* *dfrA1*	Sulfonamide, tetracycline, aminoglycoside aminoglycoside trimethoprim	Sulfamethoxazole–trimethoprim
MLW10	*S. sonnei*	9,169 *8,401*	*aadA1, sat2, dfrA1* *tet(A), aph(6)-Id, aph(3″)-Ib, sul2*	Aminoglycoside, streptothricin, trimethoprim tetracycline, aminoglycoside, sulfonamide	Sulfamethoxazole–trimethoprim
MLW11	*S. boydii*	6,768 *4,309*	*sul2, aph(3″)-Ib, dfrA14, aph(6)-Id tet(B*)	Sulfonamide, aminoglycoside, trimethoprim, aminoglycoside tetracycline	Sulfamethoxazole–trimethoprim, ampicillin
MLW12	*S. sonnei*	9,169 *8,412*	*dfrA1, sat2, aadA1* *tet(A), aph(6)-Id, aph(3″)-Ib, sul2*	Trimethoprim, streptothricin, aminoglycoside tetracycline, aminoglycoside, sulfonamide	Sulfamethoxazole–trimethoprim, ampicillin
MLW13	*S. flexneri Xv (4* c)	8,717 *7,876* *2,486*	*blaTEM-1, aph(6)-Id, aph(3″)-Ib, sul2* *tet(A) dfrA14*	Beta-lactam, aminoglycoside, sulfonamide tetracycline trimethoprim	Sulfamethoxazole–trimethoprim, ampicillin
MLW14	*S. sonnei*	9,168 *8,401*	*aadA1, sat2, dfrA1* *sul2, tet(A), aph(6)-Id, aph(3″)-Ib*	Aminoglycoside, streptothricin, trimethoprim sulfonamide, tetracycline, aminoglycoside	Sulfamethoxazole–trimethoprim
MLW15	*S. sonnei*	9,170 *8,412*	*aadA1, sat2, dfrA1* *aph(6)-Id, aph(3″)-Ib, sul2, tet(A*)	Aminoglycoside, streptothricin, trimethoprim aminoglycoside, sulfonamide, tetracycline	Sulfamethoxazole–trimethoprim
MLW16	*S. flexneri Xv (4* c)	10,626 *2,714* *2,486* *2,227*	*blaTEM-1, qnrS1* *sul2, aph(3″)-Ib, aph(6)-Id dfrA14* *tet(A*)	β-lactam, quinolone Sulfonamide, aminoglycoside trimethoprim tetracycline	Sulfamethoxazole–trimethoprim, ampicillin
MLW17	*S. boydii*	6,768 *4,309* *2,700*	*sul2, aph(6)-Id, dfrA14, aph(3″)-Ib tet(B) qnrB19*	Sulfonamide, aminoglycoside, trimethoprim, aminoglycoside tetracycline quinolone	Sulfamethoxazole–trimethoprim
MLW18	*S. sonnei*	8,401 *6,860*	*tet(A), aph(6)-Id, aph(3″)-Ib, sul2* *aadA1, sat2, dfrA1*	Tetracycline, aminoglycoside, sulfonamide aminoglycoside, streptothricin, trimethoprim	Sulfamethoxazole–trimethoprim, ampicillin
MLW19	*S. sonnei*	9,169 *8,401*	*dfrA1, sat2, aadA1* *tet(A), aph(6)-Id, aph(3″)-Ib, sul2*	Trimethoprim, streptothricin, aminoglycoside Tetracycline, aminoglycoside, sulfonamide	Sulfamethoxazole–trimethoprim
MLW20	*S. sonnei*	8,401 *6,421*	*tet(A), aph(6)-Id, aph(3″)-Ib, sul2* *aadA1, sat2, dfrA1*	Tetracycline, aminoglycoside, sulfonamide aminoglycoside, streptothricin, trimethoprim	Sulfamethoxazole–trimethoprim
MLW21	*S. boydii*	3,623 *3,092* *3,076*	*dfrA14, aph(6)-Id tet(B) aph(3″)-Ib, sul2*	Trimethoprim, aminoglycoside tetracycline aminoglycoside, sulfonamide	Sulfamethoxazole–trimethoprim
MLW22	*S. boydii*	6,768 *4,306*	*sul2, aph(3″)-Ib, dfrA14, aph(6)-Id tet(B*)	Sulfonamide, aminoglycoside, trimethoprim, aminoglycoside tetracycline	Sulfamethoxazole–trimethoprim
MLW23	*S. boydii*	6,768 *4,309* *2,700*	*sul2, aph(3″)-Ib, dfrA14, aph(6)-Id tet(B) qnrB19*	Sulfonamide, aminoglycoside, trimethoprim, aminoglycoside tetracycline quinolone	Sulfamethoxazole–trimethoprim
MLW24	*S. boydii*	6,768 *4,309*	*sul2, aph(3″)-Ib, dfrA14, aph(6)-Id tet(B*)	Sulfonamide, aminoglycoside, trimethoprim, aminoglycoside tetracycline	Sulfamethoxazole–trimethoprim
MLW25	*S. flexneri Xv (4* c)	8,717 *7,191* *2,486*	*blaTEM-1, aph(6)-Id, aph(3″)-Ib, sul2* *tet(A) dfrA14*	β-lactam, aminoglycoside, sulfonamide tetracycline trimethoprim	Sulfamethoxazole–trimethoprim, ampicillin, mecillinam
MLW26	*S. flexneri 6*	21,829 *6,199*	*catA1, dfrA7, qacEdelta1, sul1* *aph(6)-Id, aph(3″)-Ib, sul2*	Phenicol, trimethoprim, quaternary ammonium, sulfonamide aminoglycoside, sulfonamide	Sulfamethoxazole–trimethoprim
MLW27	*S. flexneri Xv (4* c)	142,287 *17,334* *2,486*	*tet(A) qnrS1, blaTEM-1, aph(6)-Id, aph(3″)-Ib, sul2* *dfrA14*	Tetracycline Quinolone, β-lactam, aminoglycoside, sulfonamide trimethoprim	Sulfamethoxazole–trimethoprim, ampicillin

*PlasmidFinder identified IncFIB(K) plasmid

There was a 100% (*n*=27/27) concordance observed between genotypic and phenotypic resistance to sulfamethoxazole–trimethoprim, as all isolates carried *dfrA14* and *sul1/sul2* resistance genes likely located on plasmids and exhibited phenotypic resistance (Table 1). Although not currently used for shigellosis treatment, the widespread presence of the resistance determinants within conserved resistance cassettes likely reflects historical selection by sulfonamide exposure and/or ongoing co-selection with other antimicrobials in this population. This concordance supports the utility of these markers in genomic AMR surveillance and reinforces concerns about the accumulation of functionally expressed MDR resistance in epidemic clones.

We did not identify any chromosomal FQR associated mutations in *gyrA*; previous studies have shown that these mutations are common in *S. sonnei* and often co-occur with resistance to unrelated antimicrobial classes [40, 41]. In Malawi, where fluoroquinolones are empirically used, such mutations could promote the persistence of MDR plasmids through genetic linkage or compensatory fitness advantages [42]. The potential for FQR-driven collateral resistance highlights the need for targeted genomic surveillance and local antimicrobial stewardship strategies.

Phenotypic testing revealed FQR, supporting the continued suitability of ciprofloxacin, a second-line empirical therapy for suspected invasive or dysentery in Malawi (Fig. 1, Table 1). Genotypically, however, 22.2% (*n*=6/27) of isolates harboured resistance determinants *qnrS1* and *qnrB19* genes. Although these determinants were not associated with phenotypic quinolone resistance in our dataset, their presence indicates genetic potential that could contribute to reduced quinolone susceptibility or future emergence of resistance under appropriate selective conditions [43]. This discordance may reflect regulatory gene silencing, low-level resistance below clinical breakpoints or compensatory mutations that delay phenotypic manifestation [44]. Alternatively, these genes may confer reduced susceptibility to quinolones, rather than clinically significant FQR [44]. While the absence of phenotypic resistance is reassuring, the presence of resistance-associated genes in one-fifth of isolates underscores the importance of genotypic and phenotypic surveillance, particularly in a region heavily reliant on ciprofloxacin.

These findings align with previous genomic surveillance studies in LMICs, in which *Shigella spp*. harbour resistance genes that are frequently associated with mobile genetic elements (MGEs) [45]. The 22.2% (*n*=6/27) fluoroquinolone genotypic resistance rate, though not yet phenotypically expressed, highlights the urgency of proactive AMR monitoring to detect early shifts in resistance patterns. Given the recognized role of horizontal gene transfer and plasmids in AMR dissemination in high-transmission *S. sonnei* and *S. flexneri* clones, targeted stewardship programmes and molecular surveillance are essential to preserve ciprofloxacin efficacy in Malawi [41].

No genotypic or phenotypic evidence of AZM resistance was detected in this study. Although this finding provides reassurance, the small sample size and single-site design limit broader inference about population-level susceptibility, which restricts broader generalizability. Furthermore, AZM is widely used in mass drug administration (MDA) programmes for trachoma elimination in endemic regions, including parts of sub-Saharan Africa, while resistance has not yet emerged in *Shigella* isolates, the sustained use of AZM in MDA creates selective pressures that could drive future resistance development in enteric pathogens [46]. Continuous surveillance is vital to detect early resistance shifts as AZM remains a cornerstone of shigellosis management.

All 27 *Shigella* isolates carried core virulence genes: *anr, iucC, iutA, nlpl, sitA, terC* and additional genes: *capU, ipaD, senB, virF* were variably distributed across serotypes (Table S5). These virulence-associated genes contribute to processes linked to host adaptation, including iron acquisition, stress response and regulation of invasion-related pathways. However, their presence alone does not confirm expression or functional contribution to disease severity [43]. Notably, *S. sonnei*, the lineage associated with recent clonal expansions and MDR, harboured the highest number of virulence genes (average of 20.1 per isolate compared with an average of 16.4 in other isolates) consistent with global epidemic potential and enhanced adaptability in human hosts (Table S5) [47]. While prior studies suggest a speculative link between virulence gene abundance and AMR accumulation, our study did not explicitly test this association [48]. Instead, the co-occurrence of extensive virulence repertoires and conserved AMR cassettes in *S. sonnei* underscores its dual threat as a high-transmission, MDR pathogen. These findings highlight the need to monitor virulence-AMR dynamics in LMIC settings, where selective pressures may favour the emergence of clones with enhanced pathogenicity and drug resistance.

### Mobile genetic elements

**Fig. 2.**
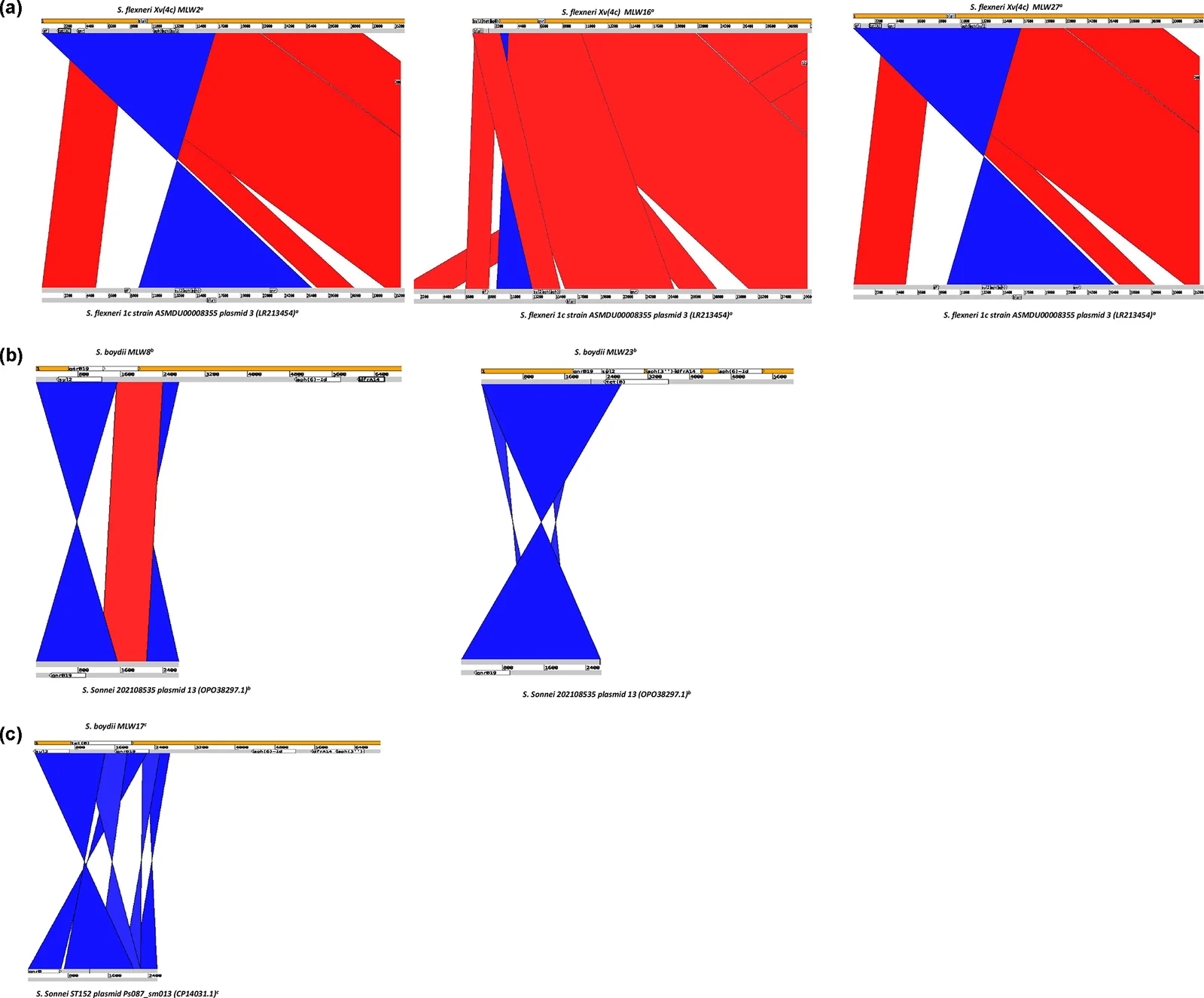
Pairwise comparison of plasmid from *Shigella* isolates causing medically attended diarrhoea in Malawian children (upper sequence) against known reference plasmids (lower sequences) (**a**) comparison against the *S. flexneri* 1 c, strain AUSMDU00008355 plasmid 3; (**b**) comparison against the *S. sonnei* strain 202108535 plasmid 13; (**c**) comparison against the *S. sonnei *ST152 plasmid ps087_sm013(cp14031.1).

To characterize the genetic context of the resistance in our isolates, we analysed AMR-associated contigs for the presence of MGEs which are key drivers of AMR dissemination [49]. We identified an IncFIB(K) plasmid replicon in one isolate (MLW2) that also contained *tet(A*) (Table 1, Table S5). However, since assemblies were generated from short-read data, we could not confirm that the resistance gene and plasmid replicon were physically located on the same genetic element. More broadly, FQR genes were detected in isolates that also carried IncFIB(K) and IncFII plasmid replicon markers in six isolates (Table S5). Of these, three *S. flexneri Xv (4 c*) isolates carried *qnrS1*, and three *S. boydii* carried *qnrB19*, with resistance-associated contigs showing similarity to previously described *Shigella* plasmids, including (LR213454) and *S. sonnei* plasmids (OPO38297.1 and CP14031.1) (Fig. 2a–b). However, due to the fragmented nature of short-read assemblies, the physical co-location of FQR and *tet(A*) genes could not be reliably determined. This is consistent with previous reports that plasmids frequently carry FQR determinants among Enterobacterales in sub-Saharan Africa and can disseminate across bacterial species and geographical regions, posing a threat to effective fluoroquinolone therapy [50, 51].

In contrast to plasmids, which are thought to contribute to the maintenance of AMR genes, transposons have shown to facilitate rapid spread of resistance across bacterial lineages [52]. Notably, the *sul2/aph* gene combination was found in phylogenetically distinct isolates. This recurrence of the same resistance gene combination across unrelated isolates suggests potential dissemination through MGEs; however, this does not demonstrate acquisition via transposons, as confirmation would require long-read sequencing or plasmid reconstruction (Fig. 1, Table 1).

These findings are consistent with potential roles for plasmids and transposons in dissemination of AMR in Malawi; however, the genomic context of these resistance determinants could not be resolved using short-read assemblies only [52]. Surveillance efforts should, therefore, monitor MGEs broadly, not only plasmids, and track selective pressures, such as antibiotic overuse. Accelerating vaccine rollout could also reduce infection rates and antibiotic demand, curbing the emergence of resistant clones [53].

## Conclusion

Our study provides crucial baseline information for public health interventions including determining antibiotic treatment suitability and supporting epidemiological understanding to inform future considerations for *Shigella* vaccines. The phylogeny highlights how regional pathogen ecology, human mobility and antimicrobial pressure collectively shape the genomic landscape of *Shigella* in Malawi. The detection of FQR-associated genes together with plasmid replicon markers, despite the absence of phenotypic resistance, identifies genetic determinants that signal a potential reservoir capable of rapidly disseminating resistance. Despite limitations in small sample size and sequencing resolution, these findings underscore the urgent need for continued genomic surveillance to detect and mitigate emerging AMR threats. A further limitation is that the use of short-read sequencing prevented reliable reconstruction of complete plasmids and the determination of the physical co-localization of AMR genes with plasmid replicons or other MGEs. Confirmation of these associations would require long-read sequencing or hybrid genome assembly.

## Supplementary material

10.1099/mgen.0.001838 Supplementary Material 1.

## Abbreviations

AMR: antimicrobial resistance
AZM: azithromycin
EFGH: Enterics for Global Health
FQR: fluoroquinolone resistance
LMICs: low- and middle-income countries
MDA: mass drug administration
MDR: multidrug-resistant
MGE: mobile genetic element
MSD: moderate-to-severe diarrhoea
NCBI: National Center for Biotechnology Information
ST: sequence type
WGSA: whole-genome sequencing analysis
WHO: World Health Organisation
